# Low-dose add-on memantine treatment may improve cognitive performance and self-reported health conditions in opioid-dependent patients undergoing methadone-maintenance-therapy

**DOI:** 10.1038/srep09708

**Published:** 2015-05-19

**Authors:** Yun-Hsuan Chang, Shiou-Lan Chen, Sheng-Yu Lee, Po See Chen, Tzu-Yun Wang, I. Hui Lee, Kao Chin Chen, Yen Kuang Yang, Jau-Shyong Hong, Ru-Band Lu

**Affiliations:** 1 Department of Psychology, Asia University, Taichung, Taiwan; 2 Division of Clinical Psychology, Institute of Allied Health Sciences, College of Medicine, National Cheng Kung University, Tainan, Taiwan; 3 Department of Psychiatry, College of Medicine, National Cheng Kung University, Tainan, Taiwan; 4 Institute of Behavioral Medicine, College of Medicine, National Cheng Kung University, Tainan, Taiwan; 5 Addiction Research Center, College of Medicine, National Cheng Kung University, Tainan, Taiwan; 6 Department of Psychiatry, National Cheng Kung University Hospital, Tainan, Taiwan; 7 Center for Neuropsychiatric Research, National Health Research Institute, Zhunan, Miaoli County, Taiwan; 8 Department of Psychiatry, National Cheng Kung University Hospital, Dou-Liou Branch, Yunlin, Taiwan; 9 Department of Psychiatry, Kaohsiung Veteran's General Hospital, Kaohsiung, Taiwan; 10 Department of Neurology, College of Medicine, Kaohsiung Medical University, Kaohsiung, Taiwan; 11 Neuropharmacology Section, Laboratory of Neurobiology, National Institute of Environmental Health Sciences/National Institutes of Health, Research Triangle Park, NC, USA

## Abstract

An important interaction between opioid and dopamine systems has been indicated, and using opioids may negatively affect cognitive functioning. Memantine, a medication for Alzheimer's disease, increasingly is being used for several disorders and maybe important for cognitive improvement. Opioid-dependent patients undergoing methadone-maintenance-therapy (MMT) and healthy controls (HCs) were recruited. Patients randomly assigned to the experimental (5 mg/day memantine (MMT+M) or placebo (MMT+P) group: 57 in MMT+M, 77 in MMT+P. Those completed the cognitive tasks at the baseline and after the 12-week treatment were analyzed. Thirty-seven age- and gender-matched HCs, and 42 MMT+P and 39 MMT+M patients were compared. The dropout rates were 49.4% in the MMT+P and 26.3% in the MMT+M. Both patient groups' cognitive performances were significantly worse than that of the HCs. After the treatment, both patient groups showed improved cognitive performance. We also found an interaction between the patient groups and time which indicated that the MMT+M group's post-treatment improvement was better than that of the MMT+P group. Memantine, previously reported as neuroprotective may attenuate chronic opioid-dependence-induced cognitive decline. Using such low dose of memantine as adjuvant treatment for improving cognitive performance in opioid dependents; the dose of memantine might be a worthy topic in future studies.

Chronic psychoactive substance users are associated with a wide variety of neuropsychological impairments, although there is no consensus on whether the impairment are permanent or temporary^1^,^2^. The chronic opioid users are associated with dysfunctional frontal networks^3^, which lead to impaired executive function and attention^4^. In addition, Baldacchnio et al. (2012)^5^ reviewed 52 articles relative to studying on how opioid affects neuropsychological function in adult patients with chronic opioid use/dependence using meta-analysis. Their analyses suggested that chronic opioid exposure is associated with impairment across variety of neuropsychological domains, including cognitive flexibility.

Current treatment for opioid dependence in practice remains controversial, although agonist maintenance using methadone or buprenorphine is the treatment of choice^6^,^7^. Methadone-maintenance-therapy (MMT) although has been suggested as effective for opioid dependence in retaining opioid-addicts in the treatment rather than buprenorphine^8^, and may ameliorate some of its cognitive deficits^9^,^10^,^11^,^12^,^13^. However, contradictory results have also been reported, viz., that patients on MMT have more neuropsychological impairments than do currently abstinent former opioid abusers^14^,^15^. Bracken et al.^16^ found that opioid-dependent patients on MMT exhibited poor performance in psychomotor speed, selective attention, and impulsivity, which implies a cognitive impairment caused by MMT. In addition, after MMT is discontinued, patients may relapse to opioid use^17^. MMT alone may not be sufficient for treating opioid dependency.

Memantine (M), a non-competitive N-methyl-D-aspartate (NMDA) receptor antagonist, has been used for more than 15 years in Europe to treat a variety of neurological diseases. Relatively recently, it has been widely prescribed (usually ≥ 20 mg/day) to treat moderate-to-severe Alzheimer's disease (AD) because of its reported benefits for people with AD^18^. Memantine, a widely recognized as NMDA antagonist^19^,^20^, but its mechanism of action is not fully understood.

Memantine at higher doses (7.5–20 mg/kg; s.c.) attenuates morphine-induced tolerance, physical dependence, and drug-seeking effects in animals^21^,^22^, its mechanism was believed to be its ability to antagonize NMDA receptors. Moreover, memantine has also benefits on people who display cognitive decline, including brain tumor^23^, Parkinson's disease^24^, alcoholism^25^, and psychiatric disorders^26^.

Memantine has been suggested to suppress the development, expression and maintenance of opioid dependence and to reduce morphine self-administration in laboratory animals^8^,^21^,^27^. Afterwards, Bisaga et al.^28^ had a pilot study to demonstrate the effect of memantine attenuating the expression of opioid withdrawal symptoms and lowering the severity of precipitated withdraw in large dosage (60 mg, PO qid) and very small sample (five opioid-addicts with cross-over design). Although, afterwards, Krupitsky et al.^29^ demonstrated that memantine significantly reduces the withdrawal symptoms in detoxified opiod-dependents and provided a rationale of using the NMDA antagonist memantine to treat opioid addicts. In their study, they used the initial dose of 10 mg/day was gradually increased to the final dose of 30 mg/day over a period of 1 week. Although, significant effective of memantine in previous studies to reduce withdrawal symptoms and severity of opioid dependence, dose-related adverse effects of memantine have been noticed, including dizziness, restlessness, headache, hallucinations, vomiting, hypertonia, and a feeling of pressure within the head^30^. Using such large dose of memantine in the previous study may be considered another issue in the future.

More recently, a relative lower-dose (0.2 mg/kg) of memantine, abolished morphine-induced conditioned-place-preference behavior in rats because of its N-methyl-D-aspartate (NMDA) receptor antagonist was reported^31^ for the neuroprotective effects. The dose condition could be a very special and highly original finding which is never reported ever for memantine. The extent of memantine dose in treating opioid-addicts remains unspecific and inconsistent, using such large dosage of memantine similar to Bisaga et al. (2001) and Krupitsky et al. (2002) may not only increase side effects but also confuse the detailed mechanism of medication. Using ultra low dosage might be better to demonstrate the specific effect of the memantine in studying its neuroprotective effect.

Since using large dose may increase side effects and might bias the mechanism of therapeutic effect of using memantine. Previously, an addict-animal model using low dose of memantine was reported the ultra-low dose effect, that addictive behavior was changed (Chen et al., unpublished data). Calculating the dosage in the animal to human, the ultra-dose of 5 mg/day memantine was applied in this study. In this study, we proposed to investigate whether a low dose of memantine would attenuate chronic opioid-induced dependent behavior and have beneficial on cognitive improvement. A double-blind, randomly stratified clinical trial with add-on low dose of memantine (5 mg/day, oral) or placebo in opioid-dependent users undergoing MMT for 12 weeks was conducted. We hypothesized that (a) chronic opioid users had worse cognitive performance compared to the health controls; (b) methadone-maintenance-therapy may delay cognitive decline; (c) methadone-maintenance-therapy add-on low-dose of memantine may improve cognitive performance compared to MMT only. To verify the effects and mechanism of memantine, a double-blind, randomly stratified clinical trial was conducted to investigate the effects of low-dose memantine on chronically opioid-dependent patients' cognitive performance and self-reported health condition undergoing MMT.

## Results

### Demographic data

**Figure 1:**
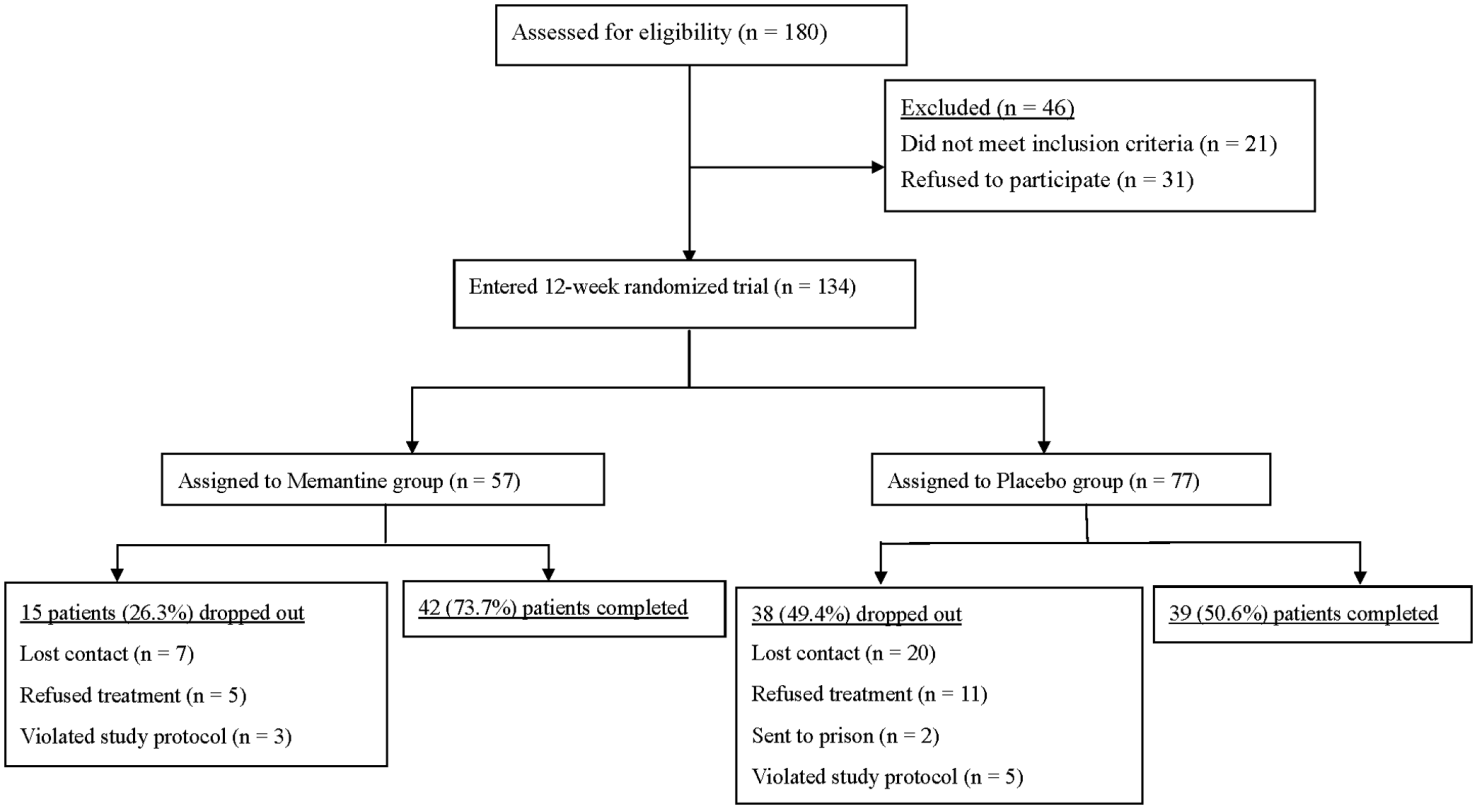
Flow chart.

At the end of this 12-week follow-up study, 81 opioid-dependent participants (MMT+P: n = 42; MMT+M: n = 39) completed tasks and their data were analyzed (Figure 1). Dropout rates of two experimental groups were 26.3% and 49.4%, respectively. The required methadone dose at baseline was not significantly different between the two MMT groups. However, a urine test revealed significantly more amphetamine users in the MMT+P group than in the MMT+M group (Table 1).

### Cognitive performance

The HCs performed significantly better than did the MMT+P and MMT+M participants on all cognitive tasks was found in the HCs (Table 2). After 12-weeks of add-on memantine treatment, a repeated measurement of variance analysis with two factors, Group with two levels (MMT+P and MMT+M) × Time with two levels (baseline and 12 weeks), was done to examine whether there was an interaction between Group and Time for each cognitive index (Table 3).

**Figure 2:**
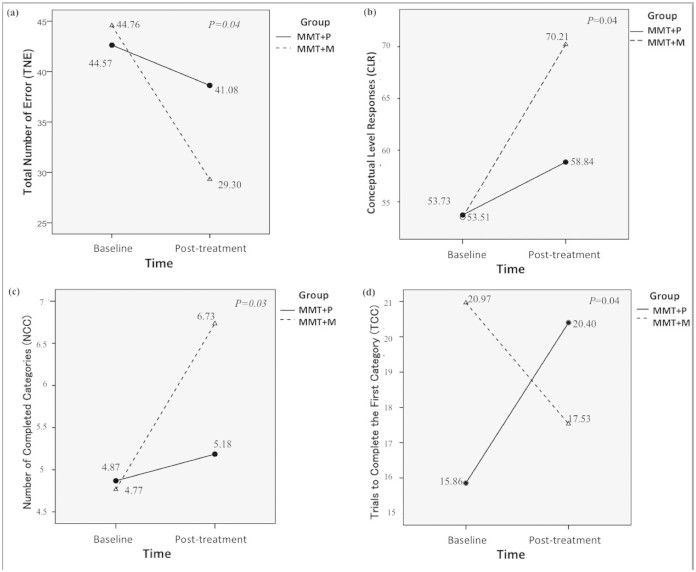
Interaction between Group × time in the numbers of total number of errors (TNE), conceptual level response (CLR), number of categories completed (NCC), and trials to complete the first category (TCC) (a) TNE, (b) CLR, (c) NCC, and (d) TCC indices of Wisconsin Card Sorting Test (indices of WCST: TNE, total number of errors; CLR, conceptual level response; NCC, number of categories completed; TCC, trials to complete the first category).

**Figure 3:**
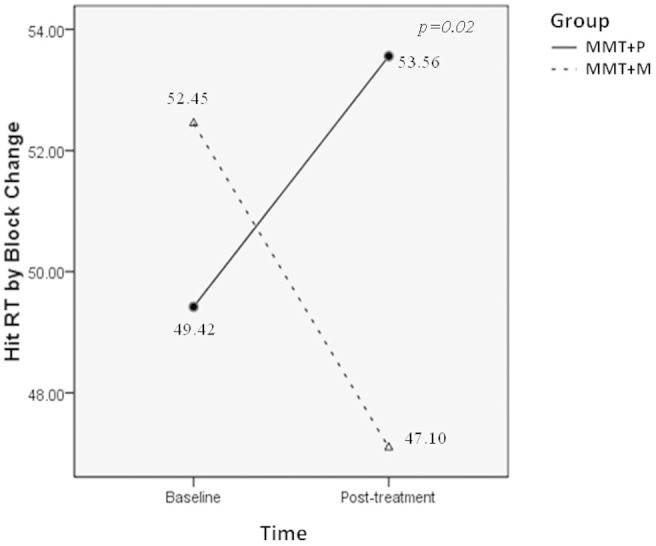
An interaction between Group and Time for the Continuous Performance Test index of HRT by Block Change.

There was a main effect of Time for WCST indices: TNE (F (1, 63) = 14.22, *p* <0.0005); CLR (F (1, 63) = 17.79, *p* <0.0005); NCC (F (1, 63) = 11.24, *p* = 0.001), as well as an interaction between Time and Group in the following indices: TNE (F (1, 63) = 4.39, *p* = 0.04); CLR (F (1, 63) = 4.16, *p* = 0.04); NCC (F (1, 63) = 5.19, *p* = 0.03); TCC (F (1,63) = 4.27, *p* = 0.04) (Figure 2 (a)–(d)). In addition, for the CPT indices, a significant main effect of Time for the HRT SE T-score (F (1, 69) = 5.89, *p* = 0.02), Variability T-score (F (1, 69) = 5.37, *p* = 0.02), HRT ISI Change T-score (F (1,69) = 4.74, *p* = 0.03), and Hit SE ISI Change T-score (F (1, 69) = 5.89, *p* = 0.02). There was an interaction between Time and Group for the HRT by Block (F (1, 69) = 5.78, *p* = 0.02) (Figure 3), but no significant main effect of Group for any cognitive performance index.

### Opiate performance index and urinary drug test

After the 12-week MMT treatment, there was no significant difference in the number between the two MMT groups (Table 4). Subsequently, McNemar's test confirmed this result determined using the OTI. The results of the urinary drug test were consistent with the OTI results, that no significantly increase of heroin or other opiate users in the MMT+M group during this 12-week treatment was found. In addition, for the criminality and health indices of the OTI, a trend of improvement was noticed, although not significant (Table 5).

## Discussion

### Cognitive performance

We found that opioid-dependent patients on MMT were cognitively impaired compared with the HCs on tests of executive function and attention^4^,^32^ related to frontal networks^3^. However, methadone is also an active agonist drug that leads to opioid dependence and opioid abuse. The MMT may cause cognitive impairment, in which previous studies have reported that patients on MMT have more neuropsychological impairments than do currently abstinent former opioid abusers^14^,^15^.

We found a larger improvement of cognitive and executive function in the MMT+M group than in the MMT+P group. Moreover, on some of the measures the improvements in the MMT+M group have been taken to the levels of HCs, indicating the effect of memantine. This finding implied an association between memantine and cognitive improvement. For the attention performance tested using CPT, however, only the HRT by Block Change index improved in the MMT+M group. This finding may indicate that memantine protects opioid-dependent patients from slower psychomotor speed and increased impulsivity caused by MMT^16^.

Although Saab et al.^33^ reported that memantine affected the cognitive flexibility in mice memory. Wesierska et al.^34^, who compared the effects of different doses of memantine on working memory training in adult male rats, reported a dose-dependent and therefore suggested that a mild NMDAR blockade using low-dose memantine would improve spatial working memory in drug-naïve rats undergoing a highly challenging task. For these inconsistent results in animal models, the causality between the therapeutic effect of memantine and cognitive domains should be investigated. Moreover, in our study, adding a low dose of memantine (5 mg/day) to MMT improved cognitive performance; therefore, a large dose (60 mg/day by Bisaga et al. (1997) and 10–30 mg/day by Krupitsky et al., 2002) used in previous studies to investigate its effectiveness in improving cognitive performance or opioid tolerance/withdrawal symptoms might not be necessary. Bisaga et al. (1997) reports treating opioid-dependent patients undergoing opiate withdrawal with memantine (37 mg/day) but do not suggest that a dose of 60 mg/kg or less is effective for treating addictive behavior. Different doses of memantine might be another topic when investigating the therapeutic effect of MMT+M on cognitive functions.

### MMT and the urinary drug test

About half of the MMT+P group (49.4%) and about one-fifth (ca. 26.3%) of the MMT+M group did not complete the tasks, which indicates a positive effect of MMT+M. A higher dropout rate in the MMT+P group than in the MMT+M group was consistent with a meta-analysis^35^ on patients with vascular dementia, which implied that memantine had a positive effect on remaining in the treatment. Because methadone is released slowly in unmetabolized metabolized form from liver and I N-demethylated in the chrome P450 enzyme, including CYP2D6 which has been reported tobe varied across different ethnics^36^,^37^,^38^. In addition, MMT program was launched by Taiwan Government in 2006 in response to the HIV/AIDS surge endemic in the Eastern Asia, and made it country widely in 2007^39^. Laio et al.^27^ conducted a study of from the Taiwan's national database of methadone service used from 2006–2008, containing 33, 549 subjects and reported that over half of the patients received methadone less than 45 mg/day and the mean dose was 46.5 ± 20.9 mg/day, which was similar to the average dose in the MMT+P group in our study. In Taiwan, the guideline suggests 5 mg increment of methadone a day for dose adjustment, MMT+M group patients had relatively lower dosage of methadone than did the MMT+P group patients after receiving 12-week MMT program further support the effect of add-on memanting in decreasing the methadone dosage as needed. At the 12-week of MMT treatment, an increase of methadone use was found in the MMT+P not in the MMT+M group, implying an effect of add-on memantine may decrease the dosage of methadone use.

The OTI data showed a decrease in criminality in the MMT+M group.

This study has some limitations. Although used to wean opioid-dependent patients from heroin and morphine, methadone itself is an addictive synthetic opioid. Whether methadone can be replaced by a non-addictive substance requires additional studies. Our total sample size (n = 81) had power> 0.8, which detected a medium effect (effect size = 0.5) for a repeated ANOVA. The dropout rate was higher in the MMT+P group than in the MMT+M group, longer follow-up duration might be needed to confirm the effect of add-on memantine in a longer-term treatment, e.g., 24 or 48 weeks, with a larger sample. The higher dropout rate was found in the MMT+ group which might influence the generalization of our results. Using per protocol analysis may bias the results, for example, participants who experienced sever adverse effect, poor financial support and family support were excluded might make the analysis reach significant level^40^. Moreover, lacking of craving data and prescription of benzodiazepines, the clinical relevance needs further study. Furthermore, because of relatively lower average methadone dose and shorter clinical experience of methadone use in Taiwan compared to other countries^6^,^41^, our finding might not be applicable to other ethnics.

## Methods

### Study design

This study, a double-blind, randomly clinical trial. The methods were carried out in "accordance" with the approved guidelines. The all experimental protocols were approved by the Institutional Review Board of National Cheng Kung University. The randomization strategy for treatment was simple randomization using excel's random number generator. Signed informed consent was obtained from all participants before they enrolled in this study. Participants who were opioid-dependent or opioid abusers were randomly assigned to one of two groups: methadone-maintenance therapy (MMT) plus add-on placebo (P) (MMT+P: MMT plus one daily placebo capsule) and methadone-maintenance therapy plus add-on memantine (M) (MMT+M: MMT plus one daily 5 mg M sustained-release capsule). Methadone dosage was increased or decreased by 5 mg when necessary in response to each participant's clinical situation. All participants were evaluated before and after the 12-week treatment.

### Participants

Opioid-dependent participants who came to the Department of Psychiatry at National Cheng Kung University Hospital were recruited. Each participant, including the healthy controls (HCs), was asked to take the Chinese version of the Mini International Neuropsychiatric Interview (MINI) to screen their psychiatric conditions and confirm that all diagnoses of opioid dependence met the DSM-IV-TR (American Psychiatric Association 2000) diagnostic criteria. The Chinese version of the MINI has been reported as reliable and has been validated^42^. All potential participants with other major and minor mental illnesses, including alcohol abuse disorder, alcohol dependence disorder, and illicit substance-use (other than heroin and morphine) disorders were excluded. All healthy controls were free of any major and minor mental illness.

The pre-MINI exclusion criteria were: being pregnant or nursing an infant; having taken any anti-inflammatory medications within 1 week before the study; having a major Axis-I DSM-IV diagnosis other than morphine dependence; and having a history of uncontrolled major physical increases in aspartate aminotransferase (AST), alanine aminotransferase (ALT), blood urea nitrogen (BUN), and creatinine levels.

Each opioid-dependent participant was given a urine drug test at each visit to determine whether they had used other substances, e.g., amphetamines or morphine, and was asked to perform neuropsychological tasks and complete the Opiate Treatment Index (OTI) interview at baseline and at the end of the 12-week follow-up. In addition, each opioid-dependent participant was required to do cognitive tasks before and after the end of this study. The HCs were required to do cognitive tasks only once—at baseline—as a control comparison. All the HCs were interviewed using the Modified Chinese Version of the Modified Schedule of Affective Disorders and Schizophrenia-Lifetime (SADS-L)^43^ to confirm that they had no mental illness or history of substance dependence or substance abuse. We recruited 134 opioid-dependent Han Chinese outpatients from the methadone therapy clinic in the Department of Psychiatry at National Cheng Kung University Hospital, and 37 age- and gender-matched HCs.

#### Wisconsin Card Sorting Test

The Wisconsin Card Sorting Test (WCST) measures the ability to perform certain types of executive functions: categorization, abstraction reasoning, maintaining sets, set switching, strategic planning, and modulating impulsive responding^44^. The test requires that certain cognitive functions be intact, for example, attention, working memory, and visual processing. Participants are required to try out different rules to find a correct method for sorting the cards. The original test used cards that had to be sorted into piles in front of four stimulus cards. The participants were instructed to infer the matching principle from the feedback provided: “correct” or “incorrect”, depending upon whether they correctly guessed the rule. The cards could be matched by number (1, 2, 3, or 4), color (yellow, green, blue, or red), or shape of the symbols (star, triangle, circle, or cross). The rule was applied for a run of trials and then changed without warning (Mei, 1998). The inter-rater liability is 0.88–0.93, within-rater reliability is 0.91–0.96, and test-retest reliability is 0.57. Performance on the WCST was scored in terms of the total number of errors (TNE), perseverative errors (PE), conceptual level responses (CLRs), number of categories completed (NCC), and trials to complete the first category (TCC).

#### Continuous Performance Test

The Conners' Continuous Performance Test (CPT)^45^ lasts for several minutes to assess the maintenance of focused attention. Optimal performance requires an adequate level of arousal, combined with an element of executive control to resist distraction and inhibit responses to stimuli resembling targets. Respondents are required to press the space bar on a computer keyboard when any letter other than “X” appears. The inter-stimulus intervals are 1, 2, and 4 seconds, with a display time of 250 ms.

The CPT produces a standard set of performance measures that include the number of errors of omission and errors of commission. (1) Errors of omission occur when the participant fails to respond to the target stimulus. (2) Errors of commission occur when the participant responds to a non-target (X) stimulus. (3) Hit reaction time (HRT) represents the Mean response time (milliseconds) for all target responses over the full six trial blocks. (4) HRT standard error (HRT SE) represents the consistency of response times and expresses the SE response to targets. (5) Detectability (*d′*) provides information on how well the examinee discriminates between targets and non-targets. (6) HRT by Block (HRT Block Change) measures changes in reaction time across the duration of the test. High scores indicate a substantial slowing in reaction times. (7) HRT Inter-Stimulus Interval (HRT ISI Change) examining changes in average reaction times across the different inter-stimulus intervals when the letters are presented at 1, 2, or 4 second intervals. According to the trade-off effect (Lachman et al., 1979), significant correlations between HRT, *d*′, and errors suggest the occurrence of a trade-off between speed and accuracy.

#### Opiate Treatment Index

The OTI is an evaluation tool for treating opiate users^46^. The original OTI has a multidimensional structure that measures six independent domains: drug use, HIV-risk-taking behavior, social functioning, criminality, health, and psychological adjustment. It has good inter-rater reliability^47^. The Chinese version of OTI contains drug use, criminality and health domains modified by Zhao et al.^48^.

### Statistical analyses

Data are means ± standard deviation. A repeated measurement with a mixed design, Group (MMT+P; MMT+M) × Time at two points during the study (pre-treatment = baseline; post-treatment = after 12 weeks of treatment) was done to determine whether there was an interaction between these sets of two factors. McNemar's test was conducted to explore changes in urine drug test values between baseline and the end of this study in each patient group. Significance was set at *p* <0.05.SPSS 18.0 was used for all statistical computations.

## Author Contributions

Y.C. wrote the draft of this manuscript and designed this study with S.C. and R.L., S.L., P.C., T.W., I.L., K.C. and Y.Y. and managed the patient recruitment and statistical analyses. This study was supervised under R.L. and J.H.

## Additional information

**Clinical Trial Registration Number:** NCT01189214 (http://clinicaltrials.gov/ct2/show/NCT01189214?id=NCT01189214&rank=1)

**How to cite this article**: Chang, Y.-H. *et al.* Low-dose add-on memantine treatment may improve cognitive performance and self-reported health conditions in opioid-dependent patients undergoing methadone-maintenance-therapy. *Sci. Rep.* **5**, 9708; DOI:10.1038/srep09708 (2015).

## Figures and Tables

**Table 1:** Demographic data

		Heroin-Dependent Patients		
	HC (n = 37)	MMT+P (n = 42)	MMT+M (n = 39)	χ^2^/F (*p*)
Gender (F/M)	31/6	36/6	34/5	0.18 (0.92)
Age (years)	36.35 ± 6.73	37.71 ± 6.57	36.24 ± 7.48	0.57 (0.57)
Duration of heroin use (years)	--	7.15 ± 6.07	7.95 ± 7.01	0.29 (0.59)
Mean methadone dose (mg/kg ± SD)	--	35.37 ± 20.04	39.73 ± 21.31	−0.93 (0.35)
		(SE = 3.13)	(SE = 3.50)	
Other substance use				
* Amphetamine (numbers)*	--	40/49	39/39	0.09 (0.77)
* Duration of amphetamine use (years)*	--	3.80 ± 4.56	3.69 ± 5.82	0.09 (0.93)
* Alcohol (numbers)*	--	11/42	12/39	0.21 (0.65)
Urine test				
* Amphetamine (+/−)*	--	9/33	2/37	4.58 (0.03)
* Morphine (+/−)*	--	24/17	22/14	0.05 (0.82)

MMT+P, methadone maintenance therapy plus placebo; MMT+M, methadone maintenance therapy plus memantine; SD, standard deviation; SE standard error.

**Table 2:** Cognitive performance comparisons at baseline

		Opioid-Dependent Participants			
		MMT+P	MMT+M		
	HC (n = 37)	(n = 42)	(n = 39)	F test (*p*)	Post hoc^a^
Wisconsin Card Sorting Test (WCST)					
* Total Number of Errors (TNE)*	30.84 ± 15.78	44.60 ± 21.85	46.47 ± 20.37	**7.14 (0.001)**	HC> MMT+P; MMT+M
* Perseverative Errors (PE)*	15.95 ± 8.78	26.48 ± 20.60	26.05 ± 17.37	**4.92 (0.009)**	HC> MMT+P; MMT+M
* Conceptual Level Responses (CLR)*	--	54.05 ± 22.33	52.24 ± 21.47	0.13 (0.72)	--
* Number of Completed Categories (NCC)*	7.30 ± 2.57	4.95 ± 2.79	4.55 ± 2.98	**10.64 (<0.0005)**	HC> MMT+P; MMT+M
* Trials to Complete the first Category (TCC)*	21.89 ± 19.34	15.41 ± 9.20	22.11 ± 16.09	2.35 (0.10)	--
Continuous Performance Test (CPT)					
* Omission T-score*	61.33 ± 33.11	61.33 ± 33.11	51.45 ± 10.75	**6.18 (0.003)**	HC> MMT+P; MMT+M> MT+P
* Commission T-score*	49.39 ± 13.72	49.39 ± 13.72	50.96 ± 13.24	0.38 (0.68)	--
* HRT T-score*	54.71 ± 15.73	54.71 ± 15.73	52.72 ± 14.56	1.69 (0.19)	--
* HRT SE T-score*	55.70 ± 18.15	55.70 ± 18.15	51.49 ± 13.71	**9.93 (<0.0005)**	HC> MMT+P; MMT+M
* Variability T-score*	49.45 ± 10.90	49.45 ± 10.90	48.14 ± 9.64	**5.72 (0.004)**	HC> MMT+P; MMT+M
* Detectability (d′) T-score*	59.67 ± 36.17	59.67 ± 36.17	58.51 ± 23.15	0.04 (0.96)	--
* Perseverations % T-score*	49.42 ± 15.23	49.42 ± 15.23	52.45 ± 12.52	3.01 (0.05)	HC> MMT+P; MMT+M
* HRT Block Change T-score*	56.93 ± 12.61	56.93 ± 12.61	58.06 ± 15.32	1.77 (0.18)	--
* HRT ISI Change T-score*	56.43 ± 15.40	56.43 ± 15.40	53.36 ± 10.91	**5.59 (0.005)**	HC> MMT+P; MMT+M
* Hit SE ISI change T-score*	61.33 ± 33.11	61.33 ± 33.11	51.45 ± 10.75	**5.58 (0.005)**	HC> MMT+P; MMT+M

HC, healthy control. HRT: Hit reaction time, representing the mean response time for all target responses;

HRT SE: Hit reaction time standard error; HRT Block Change: Hit reaction time by block;

HRT ISI Change: Hit reaction time Inter-Stimulus Interval; ^a^Fisher's least significant difference (LSD) post hoc comparison (> indicates better performance).

**Table 3:** Average scores of each cognitive task at the baseline and endpoint after follow up for two patient groups

	Baseline		Post-Treatment	
	MMT+P	MMT+M	MMT+P	MMT+M
Wisconsin Card Sorting Test (WCST)				
* Total Number of Errors (TNE)*	44.60 ± 21.85	46.47 ± 20.37	41.40 ± 21.28	31.32 ± 17.72
* Perseverative Errors (PE)*	26.48 ± 20.60	26.05 ± 17.37	21.18 ± 16.27	16.81 ± 14.38
* Conceptual Level Responses (CLR)*	53.73 ± 22.67	53.51 ± 21.90	58.84 ± 22.91	70.21 ± 15.05
* Number of Completed Categories (NCC)*	4.87 ± 2.82	4.77 ± 3.03	5.18 ± 3.45	6.73 ± 2.84
* Trials to Complete the first Category (TCC)*	15.86 ± 9.62	20.97 ± 15.69	20.40 ± 12.68	17.53 ± 13.92
Continuous Performance Test (CPT)				
* Omission T-score*	61.33 ± 33.11	51.45 ± 10.75	69.54 ± 55.94	55.33 ± 32.82
* Commission T-score*	50.13 ± 11.31	50.34 ± 10.44	50.96 ± 10.99	48.38 ± 8.50
* HRT T-score*	49.39 ± 13.72	50.96 ± 13.24	52.27 ± 13.40	53.30 ± 12.71
* HRT SE T-score*	54.71 ± 15.73	52.72 ± 14.56	61.50 ± 17.47	55.98 ± 17.42
* Variability T-score*	55.70 ± 18.15	51.49 ± 13.71	63.55 ± 18.00	57.49 ± 13.71
* Detectability (d′) T-score*	49.45 ± 10.90	48.14 ± 9.64	47.45 ± 11.18	46.29 ± 10.47
* Perseverations % T-score*	59.67 ± 36.17	58.51 ± 23.15	74.53 ± 58.52	53.08 ± 12.21
* HRT Block Change T-score*	49.42 ± 15.23	52.45 ± 12.52	53.56 ± 12.59	47.10 ± 11.07
* HRT ISI Change T-score*	56.93 ± 12.61	58.06 ± 15.32	61.93 ± 14.56	62.10 ± 15.93
* Hit SE ISI change T-score*	56.43 ± 15.40	53.36 ± 10.91	63.14 ± 15.96	59.14 ± 19.05

**Table 4:** Urine drug test for amphetamine use, morphine use, and methadone dose, and Mean Opiate Treatment Index (OTI) at baseline and after 12 weeks of treatment

	Baseline			Post-Treatment		
	MMT+P	MMT+M	χ^2^/*T* (*p*)	MMT+P	MMT+M	χ^2^/*T* (*p*)
Urine test						
* Amphetamine use (+/−)*	9/33	2/37	4.58 (0.03)	9/33	4/35	1.87 (0.17)
* Morphine use (+/−)*	24/17	22/14	0.05 (0.82)	19/21	19/15	0.52 (0.47)
Methadone dose (mg/day)	35.37 ± 20.04	39.73 ± 21.31	−0.93 (0.35)	43.38 ± 25.66	37.35 ± 22.62	1.10 (0.28)
	(SE = 3.13)	(SE = 3.50)		(SE = 4.22)	(SE = 3.58)	
Opiate Treatment Index (OTI)						
* Heroin Q score*	1.21 ± 1.30	1.05 ± 1.20	0.43	0.37 ± 0.77	0.54 ± 1.22	−0.76 (0.45)
	(SE = 0.22)	(SE = 0.21)	(0.66)	(SE = 0.13)	(SE = 0.21)	
* Opiate Q Score*	0	0	--	0	0	--
* Crime1 Q Score*	0	0.06 ± 0.35	−1.04	0	0	--
		(SE = 0.06)	(0.30)			
* Health2 Q Score*	5.42 ± 4.23	4.59 ± 4.48	0.85	4.11 ± 4.65	3.25 ± 3.14	0.90 (0.37)
	(SE = 0.71)	(SE = 0.79)	(0.40)	(SE = 0.77)	(SE = 0.56)	

^1^Criminality: number of crime offended in four crime areas: property crime, dealing, fraud, and crimes involving violence.

^2^Health: The higher the score, the poorer the overall health of the subject.

**Table 5:** McNemar's test showed no significant change in the number of opioid-dependent participants who had positive results for urinary amphetamine or morphine use at baseline and became negative

	Amphetamine/Morphine Use			
	Baseline (n)		Post-Treatment (n)	
Group	Negative	Positive	Negative	Positive
MMT+P (n = 40)	27	4	5	4
MMT+M (n = 33)	29	3	1	0

Urinary amphetamine: *P*_P_ = 1.00/*P*_M_ = 0.63; urinary morphine: *P*_P_ = 0.23/*P*_M_ = 0.61.

MMT+P, morphine maintenance therapy plus placebo; MMT+M, morphine maintenance therapy plus memantine.
