# Corrigendum: Low-dose add-on memantine treatment may improve cognitive performance and self-reported health conditions in opioid-dependent patients undergoing methadone-maintenance-therapy

**DOI:** 10.1038/srep46849

**Published:** 2017-06-15

**Authors:** Yun-Hsuan Chang, Shiou-Lan Chen, Sheng-Yu Lee, Po See Chen, Tzu-Yun Wang, I. Hui Lee, Kao Chin Chen, Yen Kuang Yang, Jau-Shyong Hong, Ru-Band Lu

Scientific Reports 5: Article number: 9708; 10.1038/srep09708 published online: 05 19 2015; updated: 06 15 2017.

In this Article, there are typographical errors in Table 2. The Continuous Performance Test (CPT) data for the MMT + P group was duplicated for the HC group. The correct Table 2 appears below as Table 1.

## Figures and Tables

**Table 1:** HC, healthy control.

	HC (n = 37)	Opioid-Dependent Participants		F test (*p*)	Post hoc^a^
		MMT + P	MMT + M		
		(n = 42)	(n = 39)		
Wisconsin Card Sorting Test (WCST)					
*Total Number of Errors* (*TNE*)	30.84 ± 15.78	44.60 ± 21.85	46.47 ± 20.37	**7.14 (0.001)**	HC > MMT + P; MMT + M
*Perseverative Errors* (*PE*)	15.95 ± 8.78	26.48 ± 20.60	26.05 ± 17.37	**4.92 (0.009)**	HC > MMT + P; MMT + M
*Conceptual Level Responses* (*CLR*)	—	54.05 ± 22.33	52.24 ± 21.47	0.13 (0.72)	—
*Number of Completed Categories* (*NCC*)	7.30 ± 2.57	4.95 ± 2.79	4.55 ± 2.98	**10.64 (<0.0005)**	HC > MMT + P; MMT + M
*Trials to Complete the first Category* (*TCC*)	21.89 ± 19.34	15.41 ± 9.20	22.11 ± 16.09	2.35 (0.10)	—
Continuous Performance Test (CPT)					
*Omission T-score*	44.76 ± 4.29	61.33 ± 33.11	51.45 ± 10.75	**6.18 (0.003)**	HC > MMT + P; MMT + M > MMT + P
*Commission T-score*	48.39 ± 9.74	50.13 ±11.31	50.34 ± 10.44	0.38 (0.68)	—
*HRT T-score*	45.65 ± 9.31	50.66 ± 14.88	49.88 ± 12.81	1.69 (0.19)	—
*HRT SE T-score*	41.82 ± 9.96	55.00 ± 15.51	53.42 ± 15.26	**9.93 (<0.0005)**	HC > MMT + P; MMT + M
*Variability T-score*	45.08 ± 8.44	56.26 ± 18.64	53.13 ± 14.78	**5.72 (0.004)**	HC > MMT + P; MMT + M
*Detectability* (*d′*) *T-score*	49.04 ± 8.78	49.18 ± 10.98	49.67 ± 10.34	0.04 (0.96)	—
*Perseverations* % *T-score*	37.65 ± 5.66	54.65 ± 28.89	57.66 ± 28.76	3.01 (0.05)	HC > MMT + P; MMT + M
*HRT Block Change T-score*	53.31 ± 6.56	50.39 ± 16.81	51.63 ± 12.06	1.77 (0.18)	—
*HRT ISI Change T-score*	47.26 ± 9.91	55.59 ± 15.24	57.17 ± 14.93	**5.59 (0.005)**	HC > MMT + P; MMT + M
*Hit SE ISI change T-score*	47.22 ± 9.97	56.76 ± 15.48	53.60 ± 11.03	**5.58 (0.005)**	HC > MMT + P; MMT + M

HRT: Hit reaction time, representing the mean response time for all target responses; HRT SE: Hit reaction time standard error; HRT Block Change: Hit reaction time by block; HRT ISI Change: Hit reaction time Inter-Stimulus Interval; ^a^Fisher’s least significant difference (LSD) post hoc comparison (> indicates better performance).

